# Supplementary material for: Crawling and Gliding: A Computational Model for Shape-Driven Cell Migration
Source: PLoS Comput Biol. 2015 Oct 21;11(10):e1004280. doi: 10.1371/journal.pcbi.1004280 (PMC4619082; doi:10.1371/journal.pcbi.1004280)
Supplement: S1 Code — (ZIP) [file pcbi.1004280.s012.zip › release/tst/doc/html/classX11Graphics.html]

Tissue Simulation Toolkit: X11Graphics Class Reference


Tissue Simulation Toolkit
 0.1.4.1


- Main Page
- Namespaces
- Classes
- Files

- Class List
- Class Hierarchy
- Class Members

Public Member Functions |
List of all members

X11Graphics Class Reference

X-Windows implementation of Graphics interface.
More...

`#include <x11graph.h>`

Inheritance diagram for X11Graphics:

[legend]

Collaboration diagram for X11Graphics:

[legend]

|  |  |
| --- | --- |
| Public Member Functions | |
|  | X11Graphics (int xfield, int yfield, const char \*movie\_file=0) |
|  | |
| virtual | ~X11Graphics (void) |
|  | |
| virtual void | BeginScene (void) |
|  | BeginScene() must be called before calling drawing functions. More... |
|  | |
| virtual void | EndScene (void) |
|  | EndScene() must be called to flush the drawing buffer and display the scene. More... |
|  | |
| void | Flush (void) |
|  | Flushes scene to window. Normally called by EndScene(). More... |
|  | |
| virtual void | Point (int color, int x, int y) |
|  | Plot a point in the Graphics window. More... |
|  | |
| virtual void | Line (int x1, int y1, int x2, int y2, int colour) |
|  | Draws a line (obviously... :-) More... |
|  | |
| void | Field (const int \*\*r, int mag=1) |
|  | Plots a field of values given by \*\*f, using color coding given by colormap file. More... |
|  | |
| virtual int | GetXYCoo (int \*X, int \*Y) |
|  | Probes the Window for user interaction, with mouse or keyboard. More... |
|  | |
| char \* | ChangeTitle (const char \*message) |
|  | Changes the title bar of the Graphics window. More... |
|  | |
| void | RecoverTitle (void) |
|  | Recovers the title prior to the last call of ChangeTitle(). More... |
|  | |
| LineType | CropSize (void) |
|  | Returns the upper left and lower right coordinates of the area occupied by cells. More... |
|  | |
| Coordinate | ReplaceBeast (Coordinate old\_size, Coordinate new\_size) |
|  | This member function was part of functionality that enables interactive resizing of the Window and CPM field, and followed by interactive replacement of the Dish's contents. The current version of CPM does not contain such functionality. More... |
|  | |
| virtual int | XField (void) const |
|  | Returns the width of the Graphics window, in pixels. More... |
|  | |
| virtual int | YField (void) const |
|  | Returns the height of the Graphics window, in pixels. More... |
|  | |
| virtual void | Write (char \*fname, int quality=-1) |
|  | Writes the Image to a file. More... |
|  | |
| void | ClearImage (void) |
|  | Clears all pixels in the Image. More... |
|  | |
| virtual void | TimeStep (void) |
|  | Implement this member function in your simulation code. More... |
|  | |
| Public Member Functions inherited from Graphics | |
| virtual | ~Graphics (void) |
|  | |

## Detailed Description

X-Windows implementation of Graphics interface.

For API see documentation of base class Graphics.

Has a number extra features: see below.

## Constructor & Destructor Documentation

|  |  |  |  |
| --- | --- | --- | --- |
| X11Graphics::X11Graphics | ( | int | *xfield*, |
|  |  | int | *yfield*, |
|  |  | const char \* | *movie\_file* = `0` |
|  | ) |  |  |

References Parameter::graphics, and Parameter::store.

|  |  |  |  |  |  |
| --- | --- | --- | --- | --- | --- |
| X11Graphics::~X11Graphics | ( | void |  | ) |  |

virtual

## Member Function Documentation

|  |  |  |  |  |  |
| --- | --- | --- | --- | --- | --- |
| void X11Graphics::BeginScene | ( | void |  | ) |  |

virtual

BeginScene() must be called before calling drawing functions.

Reimplemented from Graphics.

|  |  |  |  |  |  |
| --- | --- | --- | --- | --- | --- |
| char \* X11Graphics::ChangeTitle | ( | const char \* | *message* | ) |  |

Changes the title bar of the Graphics window.

Parameters
:   |  |  |
    | --- | --- |
    | message | Text to display in title bar. |

Referenced by RecoverTitle(), and ReplaceBeast().

|  |  |  |  |  |  |
| --- | --- | --- | --- | --- | --- |
| void X11Graphics::ClearImage | ( | void |  | ) |  |

inline

Clears all pixels in the Image.

References Point().

|  |  |  |  |  |  |
| --- | --- | --- | --- | --- | --- |
| LineType X11Graphics::CropSize | ( | void |  | ) |  |

Returns the upper left and lower right coordinates of the area occupied by cells.

Returns
:   Bounding box as a LineType structure {int x1,int y1,int x2,int y2}.

Warning: Assumes the window only displays cells (i.e. no PDE fields etc.). If you need this, better implement it as a member function of class CellularPotts.

References li::x1, li::x2, li::y1, and li::y2.

|  |  |  |  |  |  |
| --- | --- | --- | --- | --- | --- |
| void X11Graphics::EndScene | ( | void |  | ) |  |

virtual

EndScene() must be called to flush the drawing buffer and display the scene.

Reimplemented from Graphics.

References Parameter::graphics, and Parameter::storage\_stride.

Referenced by Field(), and GetXYCoo().

|  |  |  |  |
| --- | --- | --- | --- |
| void X11Graphics::Field | ( | const int \*\* | *f*, |
|  |  | int | *mag* = `1` |
|  | ) |  |  |

virtual

Plots a field of values given by \*\*f, using color coding given by colormap file.

Only implemented in X11Graphics. No checks. Usage not recommended.

Parameters
:   |  |  |
    | --- | --- |
    | f | Double pointer to array of integers, giving color indices using standard colormap ('default.ctb'). |
    | mag | magnification factor. |

Reimplemented from Graphics.

References EndScene(), Parameter::graphics, and Point().

|  |  |  |  |  |  |
| --- | --- | --- | --- | --- | --- |
| void X11Graphics::Flush | ( | void |  | ) |  |

inline

Flushes scene to window. Normally called by EndScene().

|  |  |  |  |
| --- | --- | --- | --- |
| int X11Graphics::GetXYCoo | ( | int \* | *X*, |
|  |  | int \* | *Y* |
|  | ) |  |  |

virtual

Probes the Window for user interaction, with mouse or keyboard.

This function should return immediately, and return 0 if there was no user interaction.

Parameters
:   |  |  |
    | --- | --- |
    | \*X,\*Y | Pointer where the clicked coordinate will be stored. |

Implements Graphics.

References EndScene(), Parameter::graphics, KEYBUFSIZE, MOTION, and RESIZE.

Referenced by ReplaceBeast().

|  |  |  |  |
| --- | --- | --- | --- |
| void X11Graphics::Line | ( | int | *x1*, |
|  |  | int | *y1*, |
|  |  | int | *x2*, |
|  |  | int | *y2*, |
|  |  | int | *colour* |
|  | ) |  |  |

virtual

Draws a line (obviously... :-)

Parameters
:   |  |  |
    | --- | --- |
    | x1,y1 | First coordinate pair. |
    | x2,y2 | Second coordinate pair. |
    | color | Color of the line, as given in the colormap file "default.ctb". |

Implements Graphics.

References Point(), and SWAP.

|  |  |  |  |
| --- | --- | --- | --- |
| void X11Graphics::Point | ( | int | *color*, |
|  |  | int | *x*, |
|  |  | int | *y* |
|  | ) |  |  |

virtual

Plot a point in the Graphics window.

Parameters
:   |  |  |
    | --- | --- |
    | color | Color index, as defined in colormap file "default.ctb", which should be in the same directory as the executable. |
    | x,y | Coordinate of point, in Graphics coordinates (typically twice as large as the cellular automata coordinates). |

Implements Graphics.

References Parameter::graphics.

Referenced by ClearImage(), Field(), and Line().

|  |  |  |  |  |  |
| --- | --- | --- | --- | --- | --- |
| void X11Graphics::RecoverTitle | ( | void |  | ) |  |

Recovers the title prior to the last call of ChangeTitle().

References ChangeTitle().

Referenced by ReplaceBeast().

|  |  |  |  |
| --- | --- | --- | --- |
| Coordinate X11Graphics::ReplaceBeast | ( | Coordinate | *old\_size*, |
|  |  | Coordinate | *new\_size* |
|  | ) |  |  |

This member function was part of functionality that enables interactive resizing of the Window and CPM field, and followed by interactive replacement of the Dish's contents. The current version of CPM does not contain such functionality.

References ChangeTitle(), GetXYCoo(), MOTION, RecoverTitle(), TRUE, co::x, and co::y.

|  |  |  |  |  |  |
| --- | --- | --- | --- | --- | --- |
| virtual void X11Graphics::TimeStep | ( | void |  | ) |  |

virtual

Implement this member function in your simulation code.

Include all actions that should be carried out during a simulation step, including PDE and CPM simulation steps. See the included examples (vessel.cpp, sorting.cpp) for more information.

Reimplemented from Graphics.

|  |  |  |  |
| --- | --- | --- | --- |
| void X11Graphics::Write | ( | char \* | *fname*, |
|  |  | int | *quality* = `-1` |
|  | ) |  |  |

virtual

Writes the Image to a file.

File format is inferred from file extension. Currently only PNG is supported by the X-Windows implementation; the Qt-implentation supports all formats supported by Qt.

Parameters
:   |  |  |
    | --- | --- |
    | fname | File name with standard image file extension (e.g. png). |
    | quality | Quality of JPEG images, defaults to -1 (no value provided). |

Implements Graphics.

|  |  |  |  |  |  |
| --- | --- | --- | --- | --- | --- |
| virtual int X11Graphics::XField | ( | void |  | ) | const |

inlinevirtual

Returns the width of the Graphics window, in pixels.

Reimplemented from Graphics.

|  |  |  |  |  |  |
| --- | --- | --- | --- | --- | --- |
| virtual int X11Graphics::YField | ( | void |  | ) | const |

inlinevirtual

Returns the height of the Graphics window, in pixels.

Reimplemented from Graphics.

---

The documentation for this class was generated from the following files:

- x11graph.h
- x11graph.cpp


---

Generated on Thu Aug 14 2014 22:04:01 for Tissue Simulation Toolkit by  

 1.8.6
